# Addressing international research challenges in child and adolescent mental health during global crises: experience and recommendations of the Co-SPACE international consortium

**DOI:** 10.1186/s13034-025-00918-0

**Published:** 2025-05-29

**Authors:** Jennifer McMahon, Sonja March, Martha Oakes, Wendy K. Silverman, Cathy Creswell, Arlen Rowe, Mohsen Rajabi, Simona Skripkauskaite

**Affiliations:** 1 Health Research Institute, Department of Psychology, University of Limerick, Limerick, Ireland; 2 Centre for Health Research, School of Psychology & Wellbeing, University of Southern Queensland, Ipswich, Australia; 3 Department of Psychiatry, University of Oxford, Oxford, UK; 4 Department of Experimental Psychology, University of Oxford, Oxford, UK; 5 School of Medicine, Yale University, New Haven, USA; 6 Department of Psychology, University of Bath, Bath, UK; 7 Department of Psychology, University of Tehran, Tehran, Iran

**Keywords:** COVID-19, Child, Adolescent, Family, Mental health, Research, Policy

## Abstract

During the most recent global crisis due to COVID-19 pandemic, mental health researchers globally were tasked with carrying out high-quality and responsive research to understand the changes and long-term trajectories in young people’s mental health symptoms. Comparative international longitudinal research has been recommended as a particularly promising avenue to understand pandemic impacts and facilitate global solutions. The Co-SPACE International Consortium comprises researchers from 14 sites who aimed to compare findings on the impact of the pandemic on young people and family mental health. This paper describes the process and challenges associated with the Consortium’s efforts to combine country-level data to produce global insights for research and clinical practice for the past three years. Several key challenges were identified, particularly about the conduct of international comparative research. These challenges concerned funding, ethics review, data sharing, variations in cultural and local contexts, lack of cross-culturally comparable or meaningful measures, research design, and dissemination. After considering these challenges, we provide a range of recommendations that provide a blueprint for the gathering of timely and robust evidence, the identification of global trends, the mobilisation of resources, and effective support to children and families in public health crises.

## Introduction

Public health crises due to pandemics, acts of war or terror, or natural disasters can lead to disruptions in young people’s lives in a variety of ways, including via exposure to physical or emotional threat, separation from peer and support networks, and increased familial conflict and stress [1, 2]. Indeed, the COVID-19 pandemic was associated with fluctuations in young people’s and their family’s mental health symptoms within a relatively short period of time in 2020–2021 [3–7]. The importance of exploring these fluctuations using high-quality data in a way that can directly inform policy was highlighted by public and expert panels as a research priority at the beginning of the pandemic [8].

International longitudinal research during global public health emergencies provides an opportunity to better understand mental health trajectories, inform the development of mental health support, and shape global research priority setting and policies. However, high-quality research studies that allow an understanding of young people’s mental health symptoms over time and across-borders during and as a result of the pandemic remain scarce [9]. In their editorial statement that evaluated mental health research during the pandemic, Wykes and colleagues [10] called for mental health scientists to be more creative. They suggested that researchers should expand cross-sectional data into longitudinal studies, if they can, and recommended merging data across studies to achieve a better understanding of varying risk factors as a relatively ‘easy’ solution. However, researchers in children and young people’s mental health face logistical, ethical, and methodological challenges that make such solutions less achievable than they may seem (see 9). These can be further exacerbated by attempts to work internationally due to unequal research ecosystems [11].

This paper highlights the challenges that we as a group of international child and young people’s mental health researchers and clinicians experienced in our efforts to combine individual study data across countries to explore global trajectories in young people’s mental health. We provide suggestions for researchers, research institutions, policy makers, and public and private funders to address these challenges in the future and facilitate research that can be rapidly translated into policy and practice.

## Method

### Context and participants

This paper summarises the experiences of researchers from 14 independent sites across 10 countries forming the Co-SPACE (COVID-19 Supporting Parents, Adolescents, and Children during Epidemics) International Consortium. The Co-SPACE study was launched by researchers at the University of Oxford on the 30th of March 2020, one week after the first UK national lockdown was announced. Soon after, study researchers shared survey measures with research colleagues around the world. Ten sites spanning eight countries began to conduct similar studies. Another four sites had already started to collect overlapping data independently and joined the Consortium later, introducing two additional countries (see Table 1). All fourteen sites from the Consortium collected parent-reported data and seven sites collected additional child-reported data. Most sites (*n* = 12) collected information about child mental health symptoms primarily through the Strengths and Difficulties Questionnaire [SDQ; 12,13]. Eleven sites also collected information on parent mental health from the Depression, Anxiety and Stress Scale [DASS-21; 14] and ten sites collected COVID-specific information, for example, via the Pandemic Anxiety Scale [PAS; 15,16]. Sample sizes ranged from 138 to > 30,000 across the sites with an average sample size of 4490 participants.

**Table 1:** Characteristics of the Co-SPACE international consortium studies

	Study information			Sample information			Study design		
1.	Study Name	Institution	Country	Respondents	Child age	Overall sample	Longitudinal	Testing occasions	Time periods
2.	Co-SPACE UK	University of Oxford	UK	Parents + children	4–16	9204	Y	16	Mar20-Mar23
3.	Co-SPACE Australia	University of New South Wales	Australia	Parents + children	4–17	945	Y	3	May20-Nov20
4.	Co-SPACE Denmark	Aarhus Universitet	Denmark	Parents + children	5–17	2880	Y	13	May20-Feb 21
5.	Co-SPACE Iran (iFACE)	University of Tehran	Iran	Parents	4–18	3213	Y	15	Mar20-Jul 21
6.	Co-SPACE Ireland	University of Limerick	Ireland	Parents + children	4–18	1805	Y	8	Apr20-Apr21
7.	COVID UnMasked	University of Southern Queensland	Australia	Parents	5–17	636	Y	3	May20- Dec21
8.	Yale Child Study Center	Yale University	USA	Parents	6–17	138	Y	8	Jun20-Sep21
9.	J-COSCA	Doshisha University	Japan	Parents	6–15	2456	Y	4	Nov20-Feb20
10.	Support and Social Impact Assessment for Parents, Adolescents and Children in Malaysia during COVID-19	Advanced Medical and Dental Institute, Universiti Sains Malaysia	Malaysia	Parents + children	7–12	409	N	1	May20-Jun20
11.	W & M Family Wellness Study	William & Mary	USA	Parents	5–17	656	Y	2	May20-Feb21
12.	BrightWave COVID-19	Universiteit Leiden	the Netherlands	Parents + children	9–12	60	Y	3	Apr20-Feb21
13.	Parenting in the time of COVID-19	Radboud University	the Netherlands	Parents	1–10	1156	Y	2	Apr20-Feb21
14.	COVID-19 Family Research	Bogazici University	Türkiye	Parents	4–12	1462	N	2	May20-Jun22
15.	Corona-Codomo^a^	National Center for Child Health and Development	Japan	Parents + children	0–17	> 30,000	N	7	Apr20-Dec21
					10–17	1991	Y	5	Dec20-Nov23

Studies in Bold denote original Co-SPACE studies. ^a^ Corona-Codomo ran two different arms of the study: a repeated cross-sectional design survey since April 2020 and a national longitudinal postal survey with a two-stage random sampling from December 2020

## Procedure

The authors drew on consensus building methodologies to develop a process to arrive at agreeing on the key issues pertaining to longitudinal research on child and family mental health in a global crisis [17, 18]. The resulting paper is a product of several stages of expert discussion and review within the Consortium. These stages involved (1) Lorentz workshop to discuss the issues (2) develop recommendations, (3) review stage. In December 2023, the Consortium held a five-day workshop at the Lorentz Center in Leiden, the Netherlands to compare country-level findings and discuss the ongoing and long-term implications of the pandemic for parents and young people to inform research, policy, and clinical practice [19]. Attendees (2 male; 15 female) were clinical researchers, including early- (*n* = 7), mid- (*n* = 6) and senior (*n* = 4) career researchers; joined by a local clinician and three young people to provide multi-stakeholder perspectives. Drawing on the collective experience discussed during this meeting, and through three years of attempts to maximise the combined utility of these research studies, we identified seven main areas of challenges, which we present here. A small working group was formed to bring these discussions together and formulate the associated recommendations (JMM, SS, SM, MO, WKS). This was then shared with the members of the Consortium (including those who did not attend the workshop) for comment and approval. Observations and amendments were integrated by the working group to develop the final statement.

[Table 1]

## Results and discussion

### Key areas of challenge

#### Funding inequalities

##### Insufficient focus on mental health research

During global crises like the COVID-19 pandemic, natural disasters or other events, timely and responsive funding significantly accelerates the research process, thereby facilitating rapid knowledge translation and dissemination. However, in the early onset of the COVID-19 pandemic research funds were predominantly directed towards physical health responses, such as vaccine development and healthcare, often overlooking research exploring family and child mental health. For instance, in Ireland, the government’s immediate research response focused largely on medical countermeasures and economic recovery [20], with no funding allocated to research on child and family mental health [21]. This oversight was particularly pronounced in low- and middle-income countries (LMICs), where mental health-related sectors have historically been underfunded or received minimal international support [22]. Yet, some jurisdictions swiftly launched mental health funding opportunities. For example, the UKRI Mental Health Network call resulted in several timely research projects focused on improving mental health during and beyond the pandemic in the UK [23].

##### Lack of focus on international research

Many funders prioritised national over international research, delaying globally relevant research. This meant that findings came mainly from established research networks in high-income countries like the United States or the United Kingdom. This further fed into pre-existing systemic challenges perpetuating inequities in global health research. Within the consortium, only 2 of 14 sites (one funding application) benefited from joint funding for collaborative research directed at multiple institutions simultaneously. The University of Oxford (UK) and National Center for Child Health and Development (Japan) teams received joint funding from UK Research and Innovation (UKRI) and the Japan Society for the Promotion of Science (JSPS) from February 2022. The scarcity of funding for international collaboration across the consortium hindered other members’ available capacity and, in turn, abilities to make global insights and inform solution development.

#### Problems arising due to ethics review processes

##### Timely initiation of studies

The onset of the pandemic saw an increase in the number of research studies, which in turn increased demand on ethics committees. Evidence shows that adapting ethical review processes to the circumstances of the pandemic led to a shorter review process [24] and the timely collection and combination of multi-site data [25]. Five of the 14 institutions represented in our consortium introduced a streamlined process of ethical review during the pandemic. Yet elsewhere, the urgency to understand trends in mental health clashed with the traditionally slow and arduous ethical review process. As a result, researchers faced significant hurdles in initiating timely studies. This resulted in three of the studies waiting for ethical approval for more than two months during the peak of the pandemic. Only two of the consortium sites were able to initiate projects within a week of ethical application to allow for early data capture of youths’ pandemic responses. Delays in ethical reviews in our Consortium may have been due, in part, to the sudden turn towards remote university and health services which consumed significant resources at the Institution level.

##### Informed Consent and Assent

In line with best practice, the conduct of mental health research with young people requires informed consent. However, whether this needs to be provided by parents and/or young people varies across countries and regions. For some Consortium sites (e.g., Türkiye and Iran), researchers refrained from data collection with young people directly due to concerns that demonstrating reliable and informed consent remotely would further delay the ethical review process. Some ethics committees (e.g., some jurisdictions in Australia) required parental consent that was provided in-person, or that could be confirmed verbally or via other means (rather than anonymous online consent), which often added burden and delays to ethics receipt and subsequently participant recruitment. In-person consent is particularly difficult during times of crisis where in-person contact is impossible (e.g. physical barriers due to safety) or restricted (e.g. imposed risk mitigation strategies) and requires alternative strategies to ensure integrity [26]. Data governance rules or ethics committees in other areas (e.g. Europe, Australia, and Malaysia) now require children to provide assent for their parents to provide information about them or require parents to discuss study participation with the child, even when the child does not directly participate in the study. The variation in consent requirements led to delays in recruitment and difficulties implementing a consistent methodology and sample composition (e.g. child and parent) across the international studies, further limiting global insights (particularly from the young people’s perspective).

#### Data sharing issues

##### Changes in data governance procedures

The pandemic coincided with changes to personal data processing regulations in many countries. This includes the EU and UK General Data Protection Regulation (GDPR) which now treats research data with the same stringency as any other personal data. This shift meant that even highly pseudonymised data was considered high risk due to its global reach and its links with sensitive populations and health data. This was the case despite participants’ consent that included express permissions to share their data with other researchers.

##### Variances in data governance procedures

Different countries and jurisdictions exhibit varying levels of commitment and adherence to data protection regulations, creating inconsistencies and additional obstacles in data sharing processes. For instance, the EU has 16 ‘adequacy’ decisions that formally recognise the equivalence across 16 non-EU countries’ and territories’ data protection frameworks and enable personal data sharing. In the context of our consortium, we would have expected this to ease the data sharing process between EU countries and Japan, the UK, and USA [27]. However, the USA agreement covers only commercial organisations participating in the EU-US Data Privacy Framework [28], whilst adequacy agreements between UK and Japan cover private-sector organisations only [29]. Other regions are not included in this data sharing arrangement. This means that it often falls onto the researchers or institutions supporting researchers to become familiar with differences in data protection frameworks and requirements across the countries and carry out various third-party security assessments to determine additional technical and organisational measures that need to be put in place to ensure safe handling of the participants personal data. Specialised support by participating institutions is invaluable during such processes but often under-resourced to handle the increasing administrative demands especially during the crisis.

#### Cultural and local context impacts interpretation

##### Variations in country responses to COVID-19

The Oxford Coronavirus Government Response Tracker (OxCGRT) project [30] recorded variations in global restrictions over time which provides an opportunity to situate any findings at the time within a broader context of pandemic related mitigation efforts. However, OxCGRT stringency scores can confound between-country differences in the ability to impose nation-wide restrictions with within-country adherence to specific policies. For instance, according to the OxCGRT data coding, school closures in Japan were imposed until June and only recommended afterwards.

It is worth noting that these closures were based on requests rather than legally binding measures, and most regions/schools in Japan implemented full and partial in-person schooling limits on multiple occasions during the pandemic [31]. Initially, the Japanese government requested school closures only until the start of the Spring Holidays (end of March) and did not extend this request; nevertheless, many local governments chose to prolong closures independently. As a result, 91% of schools were under temporary closure by April 22, 2020, 86% remained closed by mid-May, and 98% had reopened by June 1 [32]. These figures appear to reflect the devolved nature of school governance in Japan where nation-wide school closures could only be implemented as a last resort, with final decisions to close or stay open left to individual schools (for private schools) or local authorities (for public schools), resulting in varied practices. While collectivist norms in Japan may have contributed to higher compliance with government recommendations compared to more individualistic societies, there were still regional and school-level differences in implementation that are not reflected in the country-wide data recorded by OxCGRT [33].

Such contextual differences make the examination of school attendance and absenteeism patterns difficult, a particular interest of the Consortium and highly relevant to the wellbeing of young people both in the immediate and long-term. Individual child school attendance was recorded across some of the Co-SPACE Consortium sites, which allowed us to partially compare how in-person school attendance may have affected children across countries. Yet, positioning those insights within broader pandemic context and long-term effects was problematic even with the context specific knowledge. As an example, Box 1 provides an overview of the challenges encountered when attempting to harmonise international school absenteeism data to understand potential global trends and inform practice and policy.

**Box 1:** The challenges of studying school absenteeism during COVID-19: A case study

One issue explored by the Consortium related to the globally growing concerns of school absenteeism and whether it was linked to young people’s mental health symptoms during the pandemic. Four consortium studies (UK, Denmark, Iran, and Japan) collected data that were likely proxy indicators of future school absenteeism and were analysable for international comparison. Specifically, they asked whether young people felt comfortable returning to school following school closures. UK, Denmark, and Iran also measured whether young people had worries regarding contracting or transmitting COVID-19, school workloads and work completed (or not completed) during lockdowns, relationships/friendships and management of distress associated with the transition back to school.
On examination of the different datasets, unfortunately, the Co-SPACE UK was the only Consortium study that had the resources to consistently collect data on children’s worries about attending school and their long-term school attendance. Their data indicated that children who were not comfortable attending school in September 2020, when schools first reopened, were significantly more likely not to be attending school two years later in March 2022 (*r* =.15, *p* <.001).
We were able to answer some questions around what predicted concerns around school attendance. We used logistic regressions to further identify the children who were not comfortable with returning to school post-lockdown in 2020 across the data from the UK (*n* = 2246), Iran (*n* = 478), Denmark (*n* = 56), and in September 2021 in Japan (*n* = 1259). We found that lower household income predicted children’s discomfort in the UK (*B* = -0.53, *p* =.003), but higher comfort with returning to schools in Iran (*B* = 0.46, *p* =.017). Higher children’s mental health symptoms predicted their discomfort with returning to school in the UK (*B* = -1.29, *p* <.001) and in Denmark (*B* = -0.38, *p* =.009). Child age (*B* = -0.76, *p* <.001) and special educational needs (SEN) (*B* = -0.44, *p* =.004) only predicted discomfort with school return in the UK (this was not evaluated in Denmark due to data quality issues). In Japan, children in secondary school were less comfortable going to school compared to primary school children (OR = 0.76 [0.59, 0.98], *p* =.030). Those who felt that their family financial situation was more difficult than the previous year were also less comfortable going to school than those who did not (OR = 0.74 [0.57, 0.96], *p* =.020).
However, analysing school absenteeism data across studies posed a significant challenge due to several factors at both national- and project- level. Many participating studies had limited resources for longitudinal data collection, resulting in an inability to compare the long-term effects of initial school worries/comfort on long-term school refusal across countries. Additionally, variations in school break schedules and inconsistent attendance recording during lockdowns further complicated analysis as finding suitable timepoints for comparison was difficult. The lack of published school absenteeism data from the pandemic’s first year in many countries hindered an understanding of crisis-specific and long-term impacts, highlighting the complexities of international comparative research on school absenteeism during global crises. Data comparability across countries was also problematic due to differing definitions and measurements of absences. Some countries recorded absences while others reported attendance; many did not differentiate between types of absences, such as truancy, illness, or emotionally based school avoidance. The presence of newly arrived refugee populations during the pandemic in certain areas further complicated data interpretation, with inadequate reporting to distinguish between absence versus non-attendance as a refugee. Both government and research datasets lacked crucial details, such as reasons for absences, their severity, detailed demographics, and contextual factors, which would have provided a more comprehensive understanding and allowed greater harmonisation of data.
This indicates that the data collected by the consortium could have identified (1) trends in discomfort returning to school, (2) the most vulnerable cohorts of young people feeling discomfort returning to school, (3) household characteristics of those most uncomfortable returning to school, (4) school, pandemic, community and societal factors associated with greater discomfort returning to school, and (5) resilience factors that promoted comfort in returning to school. Identifying these trends during the early stages of the pandemic could have informed timely preventative measures for school absenteeism among those at risk. However, the abovementioned issues created a missed opportunity to perform a much more robust analysis with potentially more generalisable findings to support and/or inform recovery efforts.

#### Lack of cross-culturally comparable or meaningful measures

##### Standardised measures

Selecting suitable child and family mental health measures for cross-country comparisons is a challenge. Consortium members were restricted to measures that were accessible, translated, or validated across different cultural contexts. In this case, the Strengths and Difficulties Questionnaire [SDQ; 12,13] was widely used to provide a broad overview of social, emotional, and behavioural difficulties. Also, many measures were copyrighted or behind prohibitive paywalls, restricting their use and dissemination in international studies. A strength of the consortium was the opportunity generated to work flexibly with questionnaire owners to facilitate use across the consortium. For example, the copyright holders (Youth in Mind) of the SDQ worked with the lead research team to enable quick access to the scale across consortium members. Although this measure of broad internalising and externalising symptoms did not necessarily capture the breadth of mental health outcomes of interest, it did allow for comparison across multiple countries which was a key aim of the Consortium.

##### Contextual metrics

Any international study unavoidably has metrics that have to be adapted for different contexts. For example, our Consortium sites had to individualise demographic measures such as household income, ethnicity answer options, special educational needs categorizations. This allowed for a more accurate and acceptable assessment within each country but complicated efforts to gather consistent and reliable data. In turn, this hindered the ability to draw meaningful, generalizable conclusions about mental health trends and needs across different countries. There is a need for research to establish simple metrics relevant across countries and cultures to enable international comparisons. For example, rather than measuring income or sociodemographic status, one item or a short scale could be used to self-report relative household wealth or hardship. For adolescent self-report, the 6-item international Family Affluence Scale (FAS) has been widely validated across European and North American contexts, although its validity in lower- and middle-income countries is yet under researched [34]. In adults, Bierman et al. [35] has previously measured economic hardship with three general questions, (1) “How often in the past month did you have trouble paying the bills?” (Responses: very often, often, sometimes, rarely, and never), (2) “How often in the past month did you not have enough money to buy goods, clothes or other things your household needed?” (same responses as item 1), and (3) “How did your finances work out in the past month?” (Responses: not enough to make ends meet, barely enough to get by, just enough to make ends meet, a little money left over, and a lot of money left over). Such relative items are generally more comparable across cultures and countries than utilising demographic indices that reference unique location characteristics.

#### Research design issues

##### Data collection

Ideally in international research, data should be collected simultaneously or under conditions that are comparable across countries to ensure consistency. This was particularly challenging during the pandemic. Variations in disease incidence, coupled with the asynchronous introduction of government restrictions (e.g., border and school closures) and public health measures (e.g., social restrictions, mask wearing), resulted in unique study designs and settings. Our consortium was able to mostly overcome this and find core research design elements that overlapped across most sites. For instance, the lead organisation established the longitudinal design and monthly assessment timepoints, and as other sites joined the consortium, they assessed the feasibility of adhering to this design in their local context. Practical considerations, such as funding, meant that not all Co-SPACE sites could collect data at all the same points. For example, one Australian study collected data every three months instead, while the Ireland site collected data at 6 monthly and 2 yearly points.

#### Recruitment

Varying recruitment procedures across sites has the potential to produce different sample compositions which may impact the interpretation and translation of results. Opportunistic sampling was most feasible across Co-SPACE sites due to time pressure in the early pandemic, albeit efforts were made by some of the projects to reach priority (e.g., via clinical trial recruitment in Yale, USA) and under-represented populations (e.g., via paid panel recruitments in the UK to reach lower-income families). This limited the national representativeness of each study and thus the population level conclusions that could be drawn. However, the diversity of the data across and within the countries still permits meaningful observations and insights on group level differences in trajectories and processes of mental health changes.

#### Dissemination challenges

The dissemination of international collaborative research must also account for different publishing norms and practices across countries. In some countries within our consortium, the use of open access publishing is often a funder requirement (e.g., in the UK and Australia). In other countries, however, the use of state funds is prohibited for certain open-access publishers [36], it may require personal resources for article processing charges in others (e.g., Türkiye and Malaysia), or is impossible due to political financial sanctions (e.g., Iran). In addition, there is more nuanced mismatch across countries in terms of viewpoints on journal metrics (e.g. indexation, ranking and impact factor), where institutions have varying expectations around each measure which may pose challenges to collaborative publications.

### Recommendations

Considering the Co-SPACE Consortium’s experiences, this consensus statement outlines the following recommendations to enhance collaborative international research taking place during future crises (see Box 2 for a breakdown of recommended actions for researchers, clinicians, policy makers/funders and institutions).

**Box 2:** Recommendations for conducting international, multi-site, longitudinal studies during public health events

Recommendations	Researchers	Stakeholders (youngpeople, parents/carers, and clinicians)	Policy Makers/ Funders	Institutions
1. Establish research networks and priorities in preparation for crises	Establish research networks and multi-stakeholder, transdisciplinary consortia that include and represent young people and families. Consider special interest groups within broader networks to facilitate this.	Inform research priorities and the development of suitable measures and protocols for accessing and obtaining the perspectives of vulnerable and hard to reach groups.	Provide dedicated support for the establishment and development of international research networks centred around child and adolescent mental health.	Provide seed funding or protected researcher time to support the development and running of networks and collaborative projects. Consider potential leveraging of existing cross-institutional agreements and student exchanges.
2. Increase dedicated funding for international mental health research	Inform funding priorities and scheme types based on need and evidence.	Inform strategies for enhancing stakeholder involvement in project requirements and application review.	Establish dedicated funding streams for mental health that can be rapidly initiated and support international and longitudinal projects with clinician involvement.	Implement rapid and supportive administrative processes (e.g. grant review and contract management) to support researchers in crisis contexts.
3. Improve data sharing and governance procedures	Work with Research Institutions to identify requirements for international collaborative research agreements and internationally accepted consent and data storage practices.	Inform project governance through project-level involvement (via advisory committees or as co-investigators) to ensure research addresses real-world problems.	Provide support for costs associated with data sharing and governance.	Work with researchers to adapt existing collaborative research agreements and procedures for international projects.
4. Improve access to validated and culturally sensitive measures	Inform the identification and development of core validated measures for child and adolescent mental health applicable across cultures.	Inform relevance of mental health measures to clinical practice and development of intervention targets.	Establish dedicated funding (grants, tenders) to support projects that develop internationally relevant core set of validated measures for child and adolescent measures. Ensure accessibility of measures via open licenses or reimbursement strategies.	Support researchers in data sharing and collaborative research agreement processes required to conduct projects that establish an international set of measures. Prioritise ‘public good’ research over commercial returns.
5. Develop inclusive and robust methods (recruitment and data synchronicity strategies)	Develop recruitment strategies to allow participation from under-represented and vulnerable populations. Implement minimum data timepoints and statistical approaches to manage data synchronicity.	Assist in identification and involvement of under-represented and vulnerable groups in child and adolescent mental health research.	Prioritise projects that address data generalisability and data synchronicity. Facilitate additional support for low-resource (funds and research expertise) countries/sites.	Establish cross-institutional schemes to support mentoring of international collaborators to enhance research capacity.
6. Establish dissemination plans as priority	Establish dissemination plans prior to project commencement. Integrate clinicians and policy makers into dissemination efforts.	Inform strategies for dissemination to the public, health services, and clinicians. Partner with researchers and funders to assist in dissemination.	Provide adequate financial support or waivers to ensure equitable dissemination of research findings across countries.	Support researchers to disseminate research outcomes nationally and internationally via media and engagement teams and financial support of publication fees.

**1. Prepare in advance by establishing research networks**

Researchers should form active research networks and collaborations, both within countries to engage local researchers and between countries to foster international partnerships, which are informed by young people, parents/ carers, and clinicians, in preparation for future crises to facilitate rapid international research. This might involve setting up special interest groups within existing networks, establishing formal consortia and conducting research priority setting activities to facilitate rapid study initiation during a crisis. Networks should include researchers from LMICs and the Global South, in particular, to enhance global insights and tackle the underrepresentation of LMICs in research. Organisations such as the European Cooperation in Science and Technology (COST) or the International Science Council are well placed to develop regional and international networks that can plan to address research challenges for future crises and individual research organisations are encouraged to support (e.g. seed funding) the establishment of local research leads and networks. Through these networks and other cross-institutional arrangements, institutions and researchers should leverage opportunities for student and staff exchanges and training opportunities to further develop research skills, collaborations, and projects. For example, as an outcome of the Co-SPACE Consortium, two European researchers have engaged in research exchange placements at Australian research institutes, enabling further development of collaborations and extension of Co-SPACE projects.

**2. Increase dedicated funding for international mental health research**

Funders should provide, or increase, dedicated funding for international research into young people’s mental health during crises and world events. These funding initiatives and priorities should be informed by researchers, young people, parents/carers, and clinicians and support a focus on mental health trends over time and across countries. Such initiatives will promote the development of evidence-based interventions, along with their relevance across contexts and provide avenues to disseminate these globally. Funding should be provided at the onset of a crisis to facilitate timely research. We encourage large funders such as the World Health Organisation (WHO), Wellcome Trust and the UN to provide leadership in such initiatives and integrate researcher and clinician involvement at all stages. Such funding initiatives should consider the challenges presented here when formulating scheme objectives and requirements.

**3. Improve data sharing and governance procedures**

Universities and research institutions should work with researchers and funders to streamline data sharing and collaboration agreement processes. For example, researchers should be supported to establish data sharing agreements with collaborators that can be used for future projects and updated in line with regulatory requirements. Existing research networks and consortiums could prepare processes for shared ethics, research design, and protocol development ahead of the crises to facilitate timely culturally sensitive cross-border research. For countries or institutions with limited experience in establishing data sharing agreements and collaborative frameworks, these networks can support the co-development of agreements that comply with international protocols while effectively accommodating local ethical standards and regulatory frameworks. Agreements should include procedures for secondary data analysis of merged datasets to answer new research questions over the course of events, as well as processes for working with young people, parents/carers, and clinicians to ensure research is addressing real-world challenges. Researchers are recommended to develop consent processes that adhere to the most stringent data privacy regulations so that data can be shared across institutions/regions with differing privacy regulations.

**4. Improve access to validated and culturally sensitive measures**

Researchers, clinicians, publishers, and funders should prioritise the creation of a database of culturally validated measures related to child and adolescent mental health to ensure that researchers from different countries can quickly select measures and begin collecting data in future public health crises. This should include both broad mental health and disorder specific measures that can assist in identifying problems and provide intervention targets across different populations and cultures. Such approaches can link to existing measurement databases such as Wellcome’s Common metrics in mental health research and Clinical Outcomes in Routine Evaluation Tools [37, 38]. It is essential that these measures are informed by the challenges of mandated standardised measures [39], as well as clinicians and relevance to clinical practice. We also recommend that funding bodies require projects to produce open-source tools and develop internationally accessible measures, as well as that research institutions prioritise such research for ‘public-good’ over potential commercial returns.

**5. Develop inclusive and robust recruitment strategies and minimum data synchronicity**

To ensure the widespread applicability of findings and development of appropriate targeted intervention strategies, it is essential that future research includes typically underrepresented groups, particularly those in Global South and resource-poor communities in LMICs. Researchers should develop partnerships with relevant community and health organisations (clinicians and health services) to facilitate inclusion of populations who are typically less involved in research studies or those who experience significant vulnerability, for example, those in remote regions, with limited digital access, or living under economic, social, health, and environmental constraints. It is further essential for researchers to consider the synchronicity of data collection timepoints in advance and establish minimum common data points that are appropriate across countries. Where synchronicity with the rest of the research network cannot be achieved– due to funding challenges, infrastructural barriers, or delayed start points, researchers should consider statistical approaches that can address these gaps. For example, planned missingness (deliberately omitting some items or survey waves for some participants) can minimise study length, reduce costs, and prevent participant fatigue. Funders and institutions are encouraged to provide additional support (such as research funding and mentoring schemes) to promote data synchronicity in LMICs, enabling local researchers or those with minimal research expertise or capacity to overcome such logistical challenges in longitudinal studies.

**6. Plan ahead for dissemination**

Dissemination plans should be integrated into project proposals, funding schemes and governance arrangements by all parties. Researchers should develop research dissemination plans (e.g. journal choices, collaborator roles, involvement of clinicians) early in the collaboration process, and potentially as a requirement of funding applications. Publishers and funders should develop strategies to support equitable research dissemination, including subsidising open-access fees where researchers are not permitted or able to cover fees. Researchers are encouraged to produce lay summaries of their findings to ensure that results are widely understandable and publicly accessible to local communities and policy makers, particularly amongst those who may not typically engage with academic journals. Additionally, policy makers should also work with researchers and clinicians to develop procedures to ensure that findings are shared in real-time during a crisis. Governments will then be able to use research findings to design or refine effective strategies to support young people and families’ mental health.

## Conclusion

The COVID-19 pandemic represented an unprecedented global crisis and has underscored significant gaps in preparedness within both the research community and policy to practice frameworks. However, it also provided a critical learning opportunity for valuable insights into the challenges and shortcomings of existing systems and response mechanisms. This paper outlines key challenges experienced by the Co-SPACE consortium in conducting international longitudinal research and provides recommendations that can provide a blueprint for the gathering of timely and robust evidence, the identification of global trends, the mobilisation of resources, and effective support to children and families in public health crises. Given the likelihood of further global crises arising in the future, it is imperative to integrate these lessons into policy development, research initiatives, and institutional preparedness. By fostering a more robust and adaptive approach, societies can strengthen their capacity to mitigate the impact of future pandemics and crises ensuring a more effective, coordinated global research response.

## Data Availability

The Co-SPACE UK data are partially (parent reports only) available under safeguarded access via the UK Data Service at http://doi.org/10.5255/UKDA-SN-8900-1, reference number SN 8900. For the data from other sites included in this paper, please contact the corresponding study team.

## Abbreviations

CoSPACE—COVID-19: Supporting parents, adolescents, and children in epidemics
COST: Cooperation in science and technology
DASS: Depression, anxiety and stress scale
FAS: Family affluence scale
GDPR: General data protection regulation
JSPS: Japan society for the promotion of science
LMIC: Low- and middle-income countries
OxCGRT: Oxford coronavirus government response tracker
SDQ: Strengths and difficulties questionnaire
UKRI: United Kingdom research and innovation
UN: United Nations
WHO: World health organisation
